# Fingerprint-Based Machine Learning Approach to Identify Potent and Selective 5-HT_2B_R Ligands

**DOI:** 10.3390/molecules23051137

**Published:** 2018-05-10

**Authors:** Krzysztof Rataj, Ádám Andor Kelemen, José Brea, María Isabel Loza, Andrzej J. Bojarski, György Miklós Keserű

**Affiliations:** 1 Department of Medicinal Chemistry, Institute of Pharmacology, Polish Academy of Sciences, 12 Smętna Street, 31-343 Krakow, Poland; krzysiek.firmowy.pan@gmail.com; 2 Medicinal Chemistry Research Group, Research Centre for Natural Sciences, Hungarian Academy of Sciences, Magyar tudósok krt. 2, H1117 Budapest, Hungary; kelemen.adam@ttk.mta.hu; 3 Grupo de Investigación “BioFarma” USC, Centro de Investigación CIMUS, Planta 3ª, Avd. de Barcelona s/n, 15782 Santiago de Compostela, Spain; pepo.brea@usc.es (J.B.); mabel.loza@usc.es (M.I.L.)

**Keywords:** target selectivity, G-protein coupled receptor, 5-HT_2B_R, chemical fingerprint

## Abstract

The identification of subtype-selective GPCR (G-protein coupled receptor) ligands is a challenging task. In this study, we developed a computational protocol to find compounds with 5-HT_2B_R versus 5-HT_1B_R selectivity. Our approach employs the hierarchical combination of machine learning methods, docking, and multiple scoring methods. First, we applied machine learning tools to filter a large database of druglike compounds by the new Neighbouring Substructures Fingerprint (NSFP). This two-dimensional fingerprint contains information on the connectivity of the substructural features of a compound. Preselected subsets of the database were then subjected to docking calculations. The main indicators of compounds’ selectivity were their different interactions with the secondary binding pockets of both target proteins, while binding modes within the orthosteric binding pocket were preserved. The combined methodology of ligand-based and structure-based methods was validated prospectively, resulting in the identification of hits with nanomolar affinity and ten-fold to ten thousand-fold selectivities.

## 1. Introduction

There is an increasing need of efficacious CNS (central nervous system) drugs with reduced off-target activity that often connected to notable subtype selectivity. Modulated by one of the major neurotransmitters, serotonergic receptors play a central role in many neuropsychiatric indications [1]. A significant number of drugs was introduced to the market targeting different 5-HT subtypes ranging from 5-HT_1–7_R [2]. Rational drug design of subtype selectivity remained a challenge in many cases.

**Figure 1:**
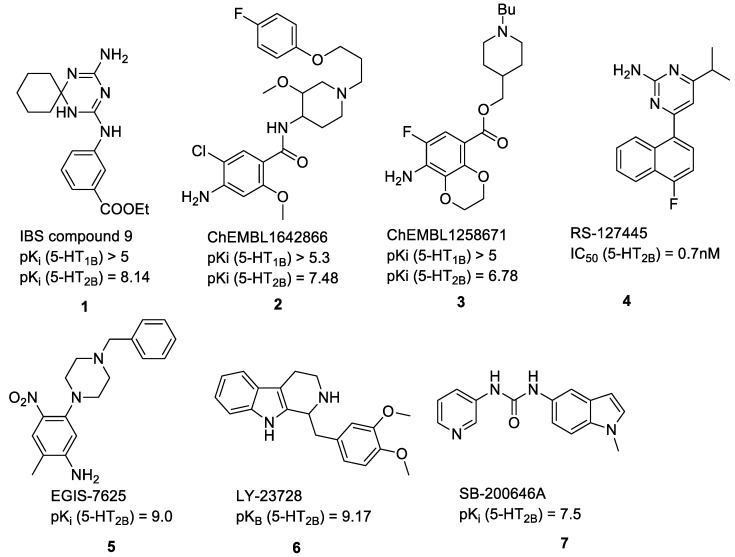
Representative 5-HT_2B_R ligands (compounds **1**–**3** with available 5-HT_1B_R and 5-HT_2B_R binding affinity data, and examples of clinical candidates **4**–**7**).

The continuously growing number of available relevant class A GPCR (G-protein coupled receptor) X-ray structures (bovine rhodopsin [3,4], β_2_AR [5], 5-HT_1B_R [6], 5-HT_2B_R [7,8,9], D_3_R [10], M_2_R [11], etc.) revealed certain important structural motifs of molecular recognition and ligand binding, including determinants of selectivity across the certain subtypes. GPCRs consist of seven transmembrane helices (and the additional intramembrane helix 8) connected in a bunch through 3-3 intracellular, and extracellular loops. Beyond the highly conserved amino acid residues localized across the helices either in the proximity of the binding pocket, or participating in the well-established DRY [12], NpxxY [13], and P-I-F motifs [14], or in the conserved disulfide bridge [15], the most conspicuous differences are present in the extracellular vestibule near the extracellular loop 2 (ECL2) region, which impacts subtype selectivity [16]. The role of the 5-HT_2B_ receptor has been implicated in a number of diseases including migraines [17,18,19], chronic heart disease [20], and irritable bowel syndrome (IBS) [21]. The chemical space of known 5-HT_2B_R ligands might be represented by a couple of chemotypes (presented in Figure 1). Examples of high affinity 5-HT_2B_R compounds showing selectivity against 5-HT_1B_R are represented by triazines (**1**) [21], piperidines (**2**, **3**), pyrimidines (e.g., RS-127445 (**4**)) [22], arylpiperazines (m-CPP [23], EGIS-7625 (**5**) [24]), tetrahydro-β-carbolines (LY-23728 (**6**), LY-272015, LY-266097) [25], and aryl ureas (SB-200646A (**7**), SB-204741, SB-215505) [26]. Interestingly, the contribution of in silico methods to the discovery of novel 5-HT_2B_R ligands is rather limited [27]. Understanding the key drivers of subtype selectivity, here we report the identification of novel selective 5-HT_2B_R ligands using a combination of ligand-based and structure-based methods.

At the first stage of screening, we applied machine learning tools [28] trained on the available structural information of known h5-HT_1B_R and h5-HT_2B_R ligands. Started from the main principle of fragment-based drug design (FBDD), these compounds were represented by Neighbouring Substructures Fingerprint (NSFP) [29], which opens the possibilities of quickly creating fragment libraries with desired target-specific, class-specific, or even family-specific properties. This new methodology is based on connections between SMARTS (SMiles ARbitrary Target Specification) patterns for finding doublets or triplets of small substructures that constitute a larger fragment. Analyzing these structural moieties, machine learning methods [30,31] are applied to recognize non-typical, activity-specific fragments for a particular target or a group of targets. Key-based substructural fingerprints depict the occurrences of a predefined set of chemical subgroups (keys) [32] within the target molecule. However, the standard key-based representations do not provide sufficient structural information. The substructures may be arranged in various ways, resulting in a vast set of possible outcomes from a single fingerprint. This may lead to ambiguities in the process of classification of active and inactive compounds, resulting in a high false positive rate. These flaws may be overcome by the addition of substructural connectivity data and combining this methodology with structure-based approaches.

A thorough analysis of the crystal structures of aminergic GPCR proteins revealed that most of the receptors have a secondary binding pocket (SBP) [16] that is formed at the extracellular part of the protein by the participation of the ECL2. This site contains a significant proportion of non-conserved amino acids across certain aminergic GPCRs that provides an opportunity to obtain subtype selectivity. Previously, we showed [33] that a docking strategy towards the SBP was able to identify bitopic compounds with improved affinity and selectivity. In our present study, we combined both methodologies, i.e., the fragment-based NSFP fingerprint with docking. Applying this approach for 2B and 1B serotonin receptors, we were able to identify new and selective 5-HT_2B_R ligands, providing potential chemical starting points for further optimization.

## 2. Results and Discussion

### 2.1. Compilation of the Training Sets for Machine Learning-Based Classification

In this study, we built a NSFP fingerprint-based machine learning model using in vitro activity data available for human 5-HT_1B_R and 5-HT_2B_R receptors in ChEMBL (biochemical database curated by the European Molecular Biology Laboratory) [34]. The compounds were divided into actives and inactives using binding affinity thresholds. Since our design concept was based on the role of the secondary binding pocket in selectivity, only compounds with 22 or more heavy atoms were considered, as they are more likely to bind to both the orthosteric binding pocket (OBP) and SBP of 5-HT_1B_R and 5-HT_2B_R.

The active/inactive sets were selected based on binding affinity recalculated from various units reported in the ChEMBL to K_i_ (actives: K_i_ ≤ 500 nM, inactives: K_i_ ≥ 1000 nM). In this way, four sets were acquired (summarized in Table 1): active for 5-HT_1B_ (1B_active_), active for 5-HT_2B_ (2B_active_), inactive for 5-HT_1B_ (1B_inactive_), and inactive for 5-HT_2B_ (2B_inactive_). 5-HT_1B_ actives and 5-HT_2B_ inactives, and 5-HT_2B_ actives and 5-HT_1B_ inactives were used for 1B_selective_ and 2B_selective_ subsets. The nonselective subset contains compounds that are active on both 5-HT_1B_ and 5-HT_2B_ receptors.

Altogether, seven training sets were used for building machine learning models (1–2. actives, 3–4. inactives, 5–6. selectives for both 1B and 2B receptor subtypes, and 7. nonselectives).

### 2.2. Building NSFP-Based Machine Learning Model

**Figure 2:**
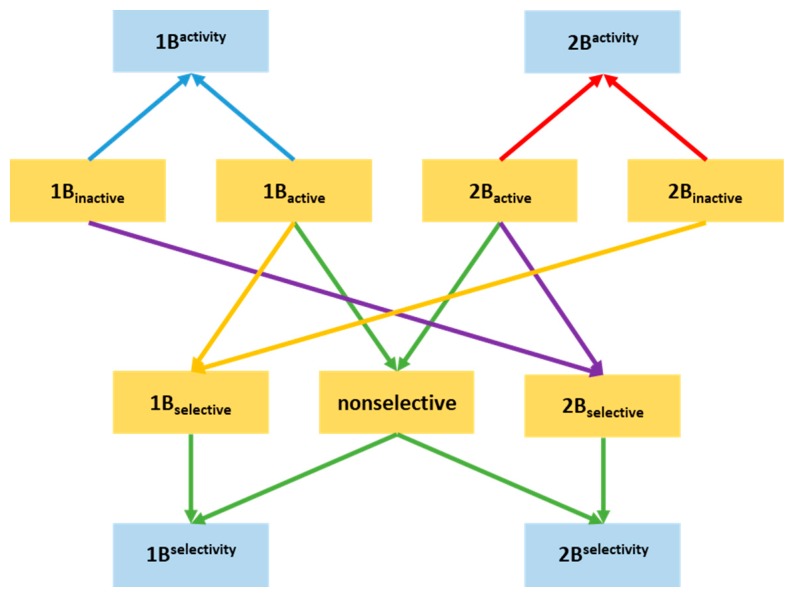
Depiction of Neighbouring Substructures Fingerprint (NSFP) representations of constructed compound sets (orange) and machine learning models (blue).

NSFP fingerprints were calculated for all of the compound sets using Klekota–Roth fingerprint (KRFP) substructure keys. A series of machine learning classifiers were created that were aimed at properly discriminating compounds. Activity classifier for 2B (2B^activity^) was built using 2B_active_ and 2B_inactive_ compounds. Selectivity classifier (2B^selectivity^) for 2B was built using 2B_selective_ and nonselective compounds in order to predict the putative selectivity of screened compounds. The same procedure was applied to 1B with its selective sets, resulting in 1B^selectivity^ and 2B^selectivity^ classifiers. Altogether, we developed two activity (for 5-HT_1B_R and 5-HT_2B_R) and two selectivity (for 5-HT_1B_R and 5-HT_2B_R) classifiers using known active, inactive, selective, and non-selective 5-HT_1B_R and 5-HT_2B_R ligands. Final models were selected from the total of 4 × 117 classifiers based on the highest acquired Matthews correlation coefficient (MCC) values. These models were used for filtering the in-stock MCule database. The summary of the model development is shown in Figure 2.

### 2.3. Prospective Machine Learning-Based Classification

First, NSFP fingerprints have also been calculated for the entire MCule database [35] of commercially available compounds. Next, each compound was classified using the four machine learning models. If a compound’s classification by 2B^activity^ was positive (compound classified as 2B active) and classification by 1B^activity^ was negative (compound classified as 1B inactive), the compound was treated as a putative 2B selective compound (^1^2B_selective_). If the opposite was true (compound classified as 1B active and 2B inactive), the compound was regarded as a putative 1B selective compound (^1^1B_selective_). Compounds achieving any other combination of classification results were disregarded from further research.

**Figure 3:**
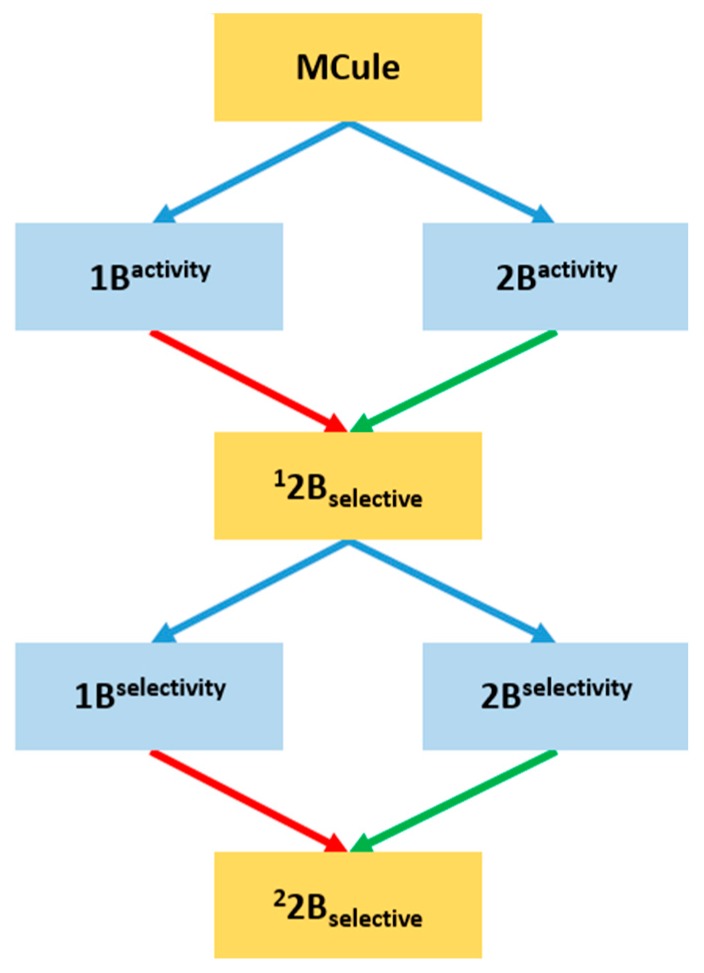
Classification procedure for putative 2B selective compounds. Green arrows represent positive classification and red arrows represent negative classification.

The second round of classification consisted of validating the compounds against the 1B^selectivity^ and 2B^selectivity^ selectivity classifiers. If a ^1^2B_selective_ compound was classified as positive by the 2B^selectivity^ and negative by the 1B^selectivity^, the compound was regarded as ^2^2B_selective_. In any other case, the compound was discarded. The summary of the prospective classification is presented in Figure 3.

**Figure 4:**
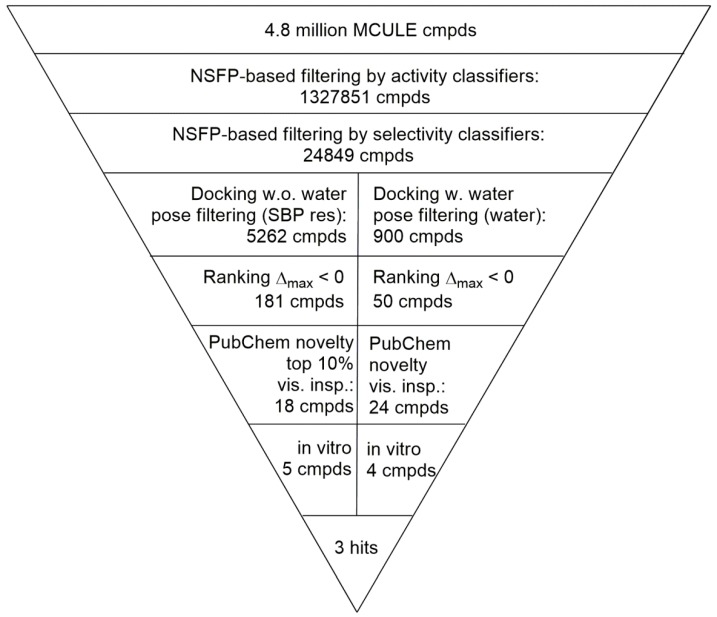
Flowchart of the virtual screening cascade used for the identification of 5-HT_2B_ selective hits.

The in-stock MCule database containing 4.8 M molecules was filtered prospectively in an activity-classification and consecutively in a selectivity-classification step resulting in 24,849 putative 5-HT_2B_ selective compounds (Figure 4).

### 2.4. Virtual Screening of the Prefiltered Set Classified by Machine Learning

**Figure 5:**
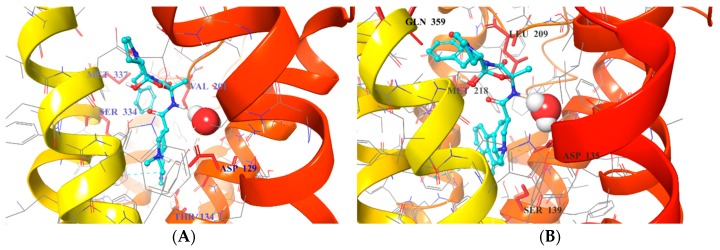
Residues (red) used in pose filtering for 5-HT_1B_R (PDB ID: 4IAQ) panel (**A**) and 5-HT_2B_R X-ray structures (PDB ID: 4IB4) panel (**B**); Water molecules forming interactions with the Asp^3.32^ shown as a sphere representation.

In the next sequential filtering step, the pre-filtered 5-HT_2B_ selective compound set of 24,849 compounds was subjected to two complementary docking workflows (i) considering non-conserved amino acid pairs and ranking-based consensus scoring; and (ii) accounting for water molecules (Figure 4). The set of 24,849 putative 5-HT_2B_ selective compounds was docked into four crystal structures available to date (PDB (Protein Data Bank) ID: 4IAQ, 4IAR [6] for h5-HT_1B_R, and 4IB4 [7], 4NC3 [8] for h5-HT_2B_R). Our docking constraints involved forming a hydrogen bond to Asp^3.32^ (Ballesteros–Weinstein numbering in superscript) [36] that anchors the charged amines of aminergic ligands [37] and any interactions with the designated non-conserved amino acid pairs present in the SBPs of the two receptors based on the work of Michino et al. [38] (see Figure 5A,B). Each pose-filtered ligand was ranked at the four receptors based on their Glide scores. The consensus scoring [39] resulted in the selection of 181 compounds showing preference to 5-HT_2B_R based on their rankings. The set of 181 ligands was sorted by their Δ_max_ values = [(RANK at 4IB4 receptor) + (RANK at 4NC3 receptor)] ‒ [(RANK at 4IAQ receptor) + (RANK at 4IAR receptor)]. The top 10% (18 ligands) were subjected to a novelty check against PubChem, and were selected for visual inspection, analyzing their binding modes. This nominated five compounds (**8**, **10**, **11**, **12**, and **13** marked with “a” in Table 2) with the largest difference of ranks obtained on 5-HT_2B_ and 5-HT_1B_ structures for in vitro tests.

As a complementary approach, we have filtered a second pool of compounds, considering the structural waters found in the X-ray structures of the receptors (PDB ID: 4IAQ [6] for h5-HT_1B_R, and 4IB4 [7] for h5-HT_2B_R). In both structures, one water molecule anchoring the oxo group of the ergoline ligands’ amide moiety with the Asp^3.32^ in TM3 was considered. Whereas the waters settle the actual position and conformation of the co-crystallized ligand, they might also govern other ligands to reach the SBP (see Figure 5). Interactions to these water molecules or directly to the protein were used for pose filtering, yielding 900 ligands with satisfactory binding modes. The 900 pose-filtered compounds were ranked, and Δ_max_ = (RANK at 4IB4 receptor) ‒ (RANK at 4IAQ receptor) values were calculated. The ligands were sorted by Δ_max_ values, and 50 structurally diverse compounds with high rank differences (i.e., Δ_max_ << 0) were selected for novelty screening. We identified 24 compounds with no known similar and active analogues in PubChem [40] that were subjected to visual inspection selecting four hits (**9**, **14**, **15**, and **16** marked with “b” in Table 2) with feasible binding mode (compounds marked with “b” in Table 2).

Altogether nine compounds that were predicted to be 2B selective were selected for competition binding assay for 5-HT_1B_R and 5-HT_2B_R (Table 2). Three hit compounds were identified (compounds **8**–**10**), showing preference towards the desired 5-HT_2B_R target over the 5-HT_1B_R off-target. Moreover, selectivity of two hit 5-HT_2B_R ligands **8** and **9** over four serotonin receptors (5-HT_1A_, 5-HT_2A_, 5-HT_6_, and 5-HT_7_) was confirmed (Table 3).

**Figure 6:**
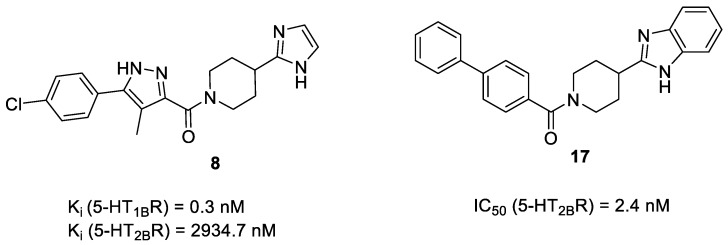
Structure of hit compound (**8**) and its closest literature analogue (**17**).

Our sequential screening protocol was able to identify potent and selective compounds with a 33% hit rate, including a highly active (subnanomolar activity) compound with almost 10^4^ selectivity factor. The 5-HT_2B_ versus 5-HT_1B_ selectivity of the closest literature analogue (compound **17** shown in Figure 6) was not confirmed by Moss et al. [41]. Lacking the relevant 5-HT_1B_ affinity, **17** was not part of the ChEMBL training set that underlines the efficiency of the NSFP-based classification models.

### 2.5. Binding Mode of Compound **8**

**Figure 7:**
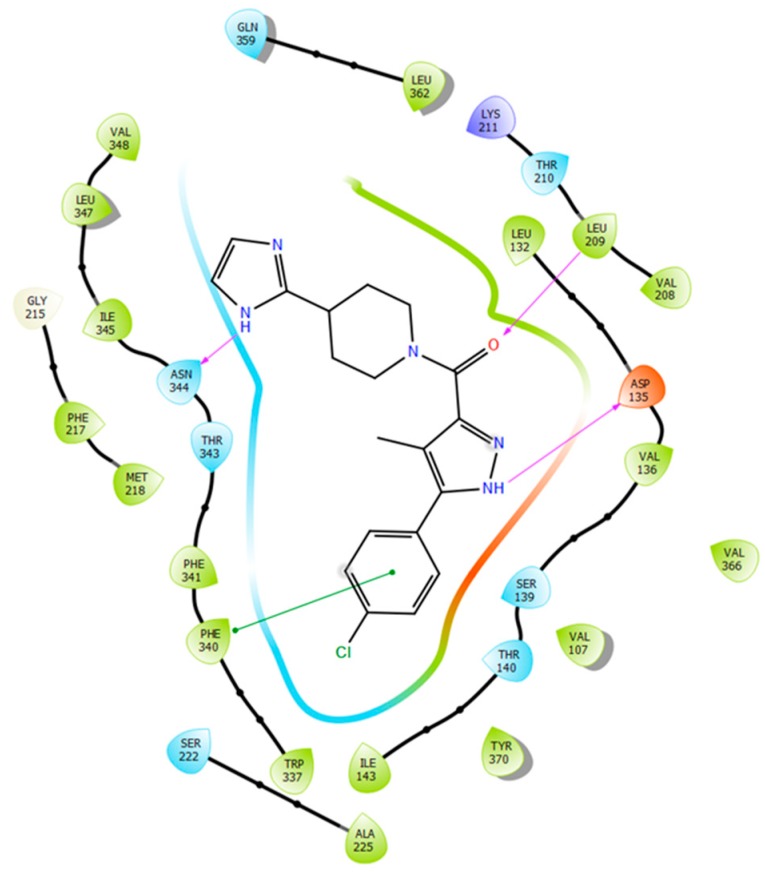
Two-dimensional (2D) interaction diagram of MCULE-7016689532 (**8**) in the 5-HT_2B_R.

The SBP of 5-HT_2B_R, which was originally occupied by the peptide portion of the ergoline ligand, is defined by several residues of ECL2, TM5, TM7, and TM6. The 4-chlorophenyl tail of **8** occupies the same orthosteric hydrophobic cavity formed by Phe340^6.51^, Phe341^6.52^, Ile143^3.40^, Trp337^6.48^, and is stacked by π–π interactions similarly to the indole ring of ergotamine (Figure 7).

**Figure 8:**
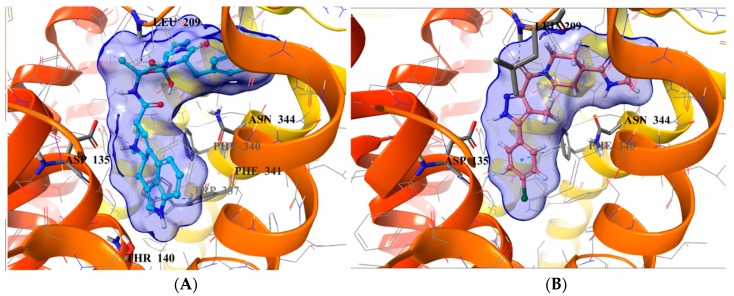
Binding site surfaces (blue spheres) of ergotamine panel (**A**) and **8** panel (**B**) in the 5-HT_2B_R crystal structure. Important ligand-contacting residues are shown in thick tube representation.

Additionally, the imidazolyl–piperidine part of the ligand is supposed to reach toward the SBP, and form a hydrogen–bond interaction with the backbone oxo group (Val208) of the same hydrophobic cleft at ECL2, such as the peptide region of ergotamine (see Figure 8). Residues Asn344^6.55^ and Met218^5.39^ are narrowing the upper chamber of the 5-HT_2B_R binding pocket, compared with 5-HT_1B_R. It has been shown [21] that disturbance of the water-network around the Asn344^6.55^ is the consequence of ligand binding to this chamber. Interestingly, the imidazole NH of the ligand formed a polar–polar interaction with this residue.

## 3. Conclusions

In this study, we aimed identifying selective 5-HT_2B_R ligands by virtual screening. Given the sequential and structural similarities of serotonergic receptors, we hypothesized that receptor selectivity is basically driven from the SBP. Consequently, we developed a screening strategy combining both ligand-based and structure-based approaches. For the first level of our hierarchical approach, we used NSFP and developed machine learning tools to select potential 5-HT_2B_R selective molecules from a large database of druglike compounds. Next, this subset was subjected to docking calculations, and we identified compounds showing different interactions with the secondary binding pockets of 5-HT_2B_R and 5-HT_1B_R. Careful analysis of the binding mode allowed us to select nine compounds for biological testing. Out of these, three compounds showed significant 5-HT_2B_R affinity, one in the low micromolar (**10**), one in the submicromolar (**9**), and one in the subnanomolar range (**8**). Compounds with submicromolar 5-HT_2B_R affinity were further profiled against a set of serotonin receptors including 5-HT_1A_R, 5-HT_2A_R, 5-HT_6_R, and 5-HT_7_R. The best compound (**8**) showed K_i_ = 0.3 nM affinity and ten thousand-fold selectivity for 5-HT_2B_R, which nominates it for in vivo testing.

## 4. Materials and Methods

### 4.1. Procedures of Machine Learning-Based Classification Model Building

Basing on the KR-NSFP representations of compounds sets, four discrimination models were built using machine learning methods: 1B^activity^ (based on 1B_active_ and 1B_inactive_ sets), 2B^activity^ (using 2B_active_ and 2B_inactive_ sets), 1B^selectivity^ (using 1B_selective_ and nonselective sets), and 2B^selectivity^ (using 2B_selective_ and nonselective sets). For each case, a series of initial models was created, using various machine learning techniques including Support Vector Machines with Radial Basis Factor and Tanimoto kernels, Naïve Bayes and Extreme Entropy Machines with Tanimoto and Sorensen kernels [30,31]. Additionally, for each method–kernel pair, a set of hyperparameters was tested, resulting in 117 initial models acquired for each final classification model. The classifiers were tested using five-fold cross-validation methodology, and for each of the compound sets, the model with the highest MCC value (see Table 4) was selected for final classification study. The entire machine learning methodology was implemented in Python programming language using scikit-learn libraries.

The equation of the MCC values calculated for the ChEMBL training set is:

$$\mathrm{MCC}\left(\mathrm{TP},\mathrm{TN},\mathrm{FP},\mathrm{FN}\right)=\frac{\mathrm{TP}\times \mathrm{TN}-\mathrm{FP}\times \mathrm{FN}}{\sqrt{\left(\mathrm{TP}+\mathrm{FP}\right)\left(\mathrm{TP}+\mathrm{FN}\right)\left(\mathrm{TN}+\mathrm{FP}\right)\left(\mathrm{TN}+\mathrm{FN}\right)}}$$

where TP—True Positive, FP—False Positive, TN—True Negative, and FN—False Negative.

### 4.2. Docking-Based Evaluation of NSFP-Classified MCule Sets

#### 4.2.1. Preparation of Ligand Sets, and Receptor Structures for Docking

Compounds used for selective ligand discovery were minimized using Schrödinger’s LigPrep algorithm [42], with the pH set to 7.4 and the number of stereoisomers set to 1. For this study, crystal structures of 5-HT_1B_R and 5-HT_2B_R were extracted from the PDB database [43] (PDB ID: 4IAQ and 4IAR [6] for 1B, and 4IB4 [7] and 4NC3 [8] for 2B). Proteins were prepared using PrepWiz software (version 2017-4) from Schrödinger [44] and transformed into a receptor grid using Schrödinger software package [45], with the binding pocket center set to the Asp^3.32^ residue. All of the docking procedures have been conducted using Schrödinger’s Glide software [46], with precision set to standard precision (SP) and varying the number of reported docking poses (5 or 10).

#### 4.2.2. Evaluation of the Docking Results

The ^2^2B_selective_ set was docked to the crystal structures of both 5-HT_1B_ and 5-HT_2B_ receptors (PDB ID: 4IAQ, 4IAR for 5-HT_1B_R and 4IB4, 4NC3 for 5-HT_2B_R, respectively), and the number of reported poses was set to 10. Additionally, poses were filtered for interactions with the SBP characteristic amino acids (see Table 5).

Scoring of reported poses was performed using custom scoring function [39], taking into consideration the rankings of compounds based on the assigned Glide docking score. It compares the numerical sum of the ranks of all of the poses acquired by a compound in both crystals of the target protein with those found in both crystals of the antitarget:

Δ_max_ = [∑(rank_target_) − ∑(rank_antitarget_)]

Since better rankings have lower numerical values (a pose with rank 1 is better than a pose with rank 5), if the compound’s Δ_max_ < 0, it was considered target-selective. For further research, the compounds with the lowest Δ_max_ were considered, as the difference in ranks between target and antitarget were the highest. Finally, the docking binding modes of the top 10% (18 ligands) were visually inspected, and five compounds (**8**, **10**, **11**, **12**, and **13** marked with “a” in Table 2) were hand-picked for in vitro validation.

The second approach included screening for interactions with water molecules buried within the binding pockets of target receptors. Of all four crystal structures of the receptors, only two contained water within the three-dimensional (3D) model: 4IAQ (1B) [6] and 4IB4 (2B) [7]. Therefore, these two structures were used in a second docking study conducted with constraints set for either a hydrogen bond/salt bridge to Asp^3.32^, or a hydrogen bond with water 2020 (4IAQ) or 2004 (4IB4). Docked compounds were filtered for proper interactions with the water molecule/Asp^3.32^ residue, and their specificity. The binding mode of the filtered compounds was checked manually, and they were subjected to a novelty check by PubChem [40] before testing with the NSFP.

The 900 pose-filtered docked ligands were first clustered by structural similarity, and a diverse set of 50 cluster centroids were subjected to novelty check using the PubChem engine. Compounds having no similar compounds or no tested analogues, or having tested but inactive analogues, were prioritized. This process yielded 24 ligands in total, out of which four compounds were selected (**9**, **14**, **15**, and **16**, marked with “b” in Table 2) after visual inspection for in vitro testing.

### 4.3. Procedures of In Vitro Screening Assays

All of the tested compounds were purchased via MCule, Inc. (Palo Alto, CA, USA). The compounds were tested for their ability to displace radioligand from the membrane of 5-HT_1B_R and 5-HT_2B_R-expressing cells. The first test was performed with a 10-µM concentration of a compound, and the inhibition percentage was calculated. If the value was above 50%, a full affinity screening was performed, and the data was gathered as K_i_ with units reported as nM.

#### 4.3.1. Competition Binding in Human 5-HT_1B_ Receptor

Serotonin 5-HT_1B_R competition binding experiments were carried out in a polypropylene 96-well plate. In each well, 5 μg of membranes from a Hela-5-HT_1B_ cell line that was prepared in our laboratory (Lot: A001/14-11-2011, protein concentration = 3179 μg/mL), 1.5 nM [^3^H]-GR125743 (83.9 Ci/mmol, 0.1 mCi/mL, Perkin Elmer NET1172100UC, K_D_ = 0.74 nM), the studied compounds and the reference compound were incubated. Non-specific binding was determined in the presence of GR55562 10 μM (TOCRIS 1054), and total binding was determined in the absence of any unlabeled compound. The reaction mixture (Vt: 250 μL/well) was incubated at 25 °C for 90 min. Then, 200 μL was transferred to a GF/C 96-well plate (Millipore, Madrid, Spain) pretreated with 0.5% of PEI and treated with binding buffer (Tris-HCl 50 mM, EDTA 1 mM, MgCl_2_ 10 mM, pH = 7.4). Afterwards, it was filtered and washed four times with 250 μL of wash buffer (Tris-HCl 50 mM, pH = 7.4), before measuring in a microplate beta scintillation counter (Microbeta Trilux, PerkinElmer, Madrid, Spain). 5-carboxytryptamine was included as a reference compound in all of the assays. Compounds were first tested at 10 micromolar; those compounds showing a percentage of displacement of specific binding higher than 50% were classified as active compounds, and K_i_ values were determined by means of concentration-response curves.

#### 4.3.2. Competition Binding in Human 5-HT_2B_ Receptor

Serotonin 5-HT_2B_R competition binding experiments were carried out in a polypropylene 96-well plate. In each well, 5 μg of membranes from a CHO-5-HT_2B_ cell line prepared in our laboratory (Lot: A003/27-03-2012, protein concentration = 3431 μg/mL), 1 nM [^3^H]-LSD (82.4 Ci/mmol, 1 mCi/mL, PerkinElmer NET638250UC, K_D_ = 0.57 nM), the studied compounds and the reference compound were incubated. Non-specific binding was determined in the presence of 5-HT 50 μM (Sigma H9523), and total binding was determined in the absence of any unlabeled compound. The reaction mixture (Vt: 250 μL/well) was incubated at 37 °C for 30 min. Then, 200 μL was transferred to a GF/C 96-well plate (Millipore, Madrid, Spain) pretreated with 0.5% of PEI and treated with binding buffer (Tris-HCl 50 mM, Ascorbic acid 0.1%, CaCl_2_ 4 mM, pH = 7.4). Afterwards, it was filtered and washed four times with 250 μL of wash buffer (Tris-HCl 50 mM, pH = 7.4) before measuring in a microplate beta scintillation counter (Microbeta Trilux, PerkinElmer, Madrid, Spain). Methysergide was included as a reference compound in all of the assays. Compounds were first tested at 10 micromolar, and those compounds showing a percentage of displacement of specific binding higher than 50% were classified as active compounds, and K_i_ values were determined by means of concentration-response curves.

#### 4.3.3. Competition Binding in Human 5-HT_1A_, 5-HT_2A_ 5-HT_6_ and 5-HT_7_ Receptors

HEK293 cells with stable expression of human serotonin 5-HT_1A_R, 5-HT_2A_, 5-HT_6_, or 5-HT_7b_ receptor (all prepared with the use of Lipofectamine 2000) were maintained at 37 °C in a humidified atmosphere with 5% CO_2_, and were grown in Dulbeco’s Modifier Eagle Medium containing 10% dialyzed fetal bovine serum and 500 mg/mL G418 sulfate. For the preparation of membranes, cells were subcultured in 10-cm diameter dishes, grown to 90% confluence, washed twice with prewarmed to 37 °C phosphate-buffered saline (PBS), and were pelleted by centrifugation (200 g) in PBS containing 0.1 mM of EDTA and 1 mM of dithiothreitol. Prior to membrane preparations, pellets were stored at −80 °C.

Cell pellets were thawed and homogenized in 20 volumes of assay buffer using an Ultra Turrax tissue homogenizer and centrifuged twice at 35,000 g for 20 min at 4 °C, with incubation for 15 min at 37 °C in between. The composition of the assay buffers was as follows: for 5-HT_1A_R: 50 mM Tris-HCl, 0.1 mM EDTA, 4 mM MgCl_2_, 10 µM pargyline, and 0.1% ascorbate; for 5-HT_2A_R: 50 mM Tris-HCl, 0.1 mM EDTA, 4 mM MgCl_2_, and 0.1% ascorbate; for 5-HT_6_R: 50 mM Tris-HCl, 0.5 mM EDTA, and 4 mM MgCl_2_, for 5-HT_7b_R: 50 mM Tris-HCl, 4 mM MgCl_2_, 10 µM pargyline, and 0.1% ascorbate. All of the assays were incubated in a total volume of 200 µL in 96-well microtiter plates for 1 h at 37 °C, except for 5-HT_1A_R and 5-HT_2A_R, which were incubated at room temperature for 1 h and 1.5 h, respectively. The process of equilibration was terminated by rapid filtration through Unifilter plates with a 96-well cell harvester, and the radioactivity retained on the filters was quantified on a Microbeta plate reader (PerkinElmer, Waltham, MA, USA).

For displacement studies, the assay samples contained as radioligands: 1.5 nM of [^3^H]-8-OH-DPAT (135.2 Ci/mmol, K_D_ = 1.5 nM) for 5-HT_1A_R; 2 nM of [^3^H]-Ketanserin (53.4 Ci/mmol, K_D_ = 2 nM) for 5-HT_2A_R; 2 nM of [^3^H]-LSD (83.6 Ci/mmol, K_D_ = 2 nM) for 5-HT_6_R, or 0.6 nM of [^3^H]-5-CT (39.2 Ci/mmol, K_D_ = 0.6 nM) for 5-HT_7_R. Non-specific binding is defined with 10 µM of 5-HT in 5-HT_1A_R and 5-HT_7_R binding experiments, whereas 10 µM of chlorpromazine or 10 µM of methiothepin were used in 5-HT_2A_R and 5-HT_6_R assays, respectively. Each compound was tested in triplicate at seven concentrations (10^−10^–10^−4^ M). Reference compounds for 5-HT_1A_R: buspirone K_i_ = 32.2 nM ± 2.9; for 5-HT_2A_R: olanzapine K_i_ = 5.6 nM ± 1.1; for 5-HT_6_R: olanzapine K_i_ = 8.8 nM ± 1.3; and for 5-HT_7_R: 5-CT K_i_ = 0.8 nM ± 0.2. The inhibition constants (K_i_) were calculated from the Cheng-Prusoff equation [47]. Results were expressed as means of at least two separate experiments.

## Figures and Tables

**Table 1:** Active, inactive, and selective 5-HT_1B_ and 5-HT_2B_ ligands retrieved from the ChEMBL with at least 22 heavy atoms.

Receptor	Number of Actives ^1^	Number of Inactives ^2^	Number of Selectives ^3^
5-HT_1B_	858 (1011)	339 (477)	86
5-HT_2B_	478 (718)	259 (351)	33

^1^ K_i_ ≤ 500 nM; ^2^ K_i_ ≥ 1000 nM; ^3^ Classified as 5-HT_2B_ selective for being active at 5-HT_2B_R, and inactive at 5-HT_1B_R; numbers in parenthesis are the total number of all actives without size-filtering.

**Table 2:** Structures and measured in vitro assay data of top-ranked compounds (sorted by percentage of inhibition and K_i_ (nM) in h5-HT_2B_R competition binding assay). Values represent the mean ± SD of three independent assays with duplicate measurements.

Compound	ID	5-HT_1B_ (%) ^1^	5-HT_2B_ (%) ^1^	5-HT_1B_ (nM) ^2^	5-HT_2B_ (nM) ^2^
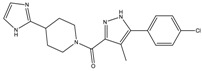	**8** ^a^	-	-	2934.7 ± 321.3	0.3 ± 0.07
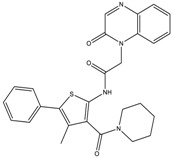	**9** ^b^	-	-	2099 ± 622.2	235.1 ± 15.9
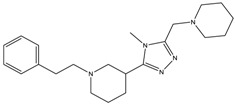	**10** ^a^	7 ± 4	-	-	2612.9 ± 422.1
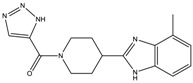	**11** ^a^	12 ± 4	40 ± 3	-	-
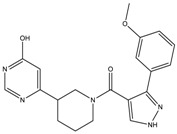	**12** ^a^	7 ± 1	36 ± 4	-	-
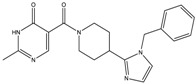	**13** ^a^	19 ± 3	34 ± 4	-	-
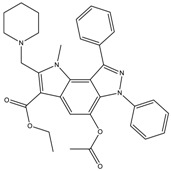	**14** ^b^	26 ± 4	34 ± 4	-	-
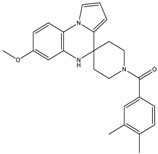	**15** ^b^	28 ± 3	31 ± 4	-	-
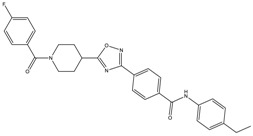	**16** ^b^	1 ± 1	24 ± 4	-	-

^1^ Percentage of inhibition measured at 10 µM concentration; ^2^ K_i_ measured (reported in nM units) if percentage of inhibition exceeded 50%; ^a^ Selection not accounting waters, ^b^ Selection accounting waters.

**Table 3:** 5-HT panel screening of the best hits. Values represent the mean ± SD of three independent assays with duplicate measurements.

Compound	ID	5-HT_1A_ K_i_ (µM)	5-HT_2A_ K_i_ (µM)	5-HT_6_ K_i_ (µM)	5-HT_7_ K_i_ (µM)
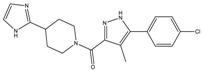	**8**	>20	>20	13.2 ± 1.5	>20
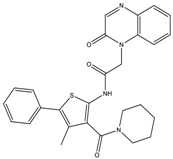	**9**	>20	>20	7.5 ± 0.9	>20

**Table 4:** Performance data of the activity and selectivity filters obtained on ChEMBL training set.

Receptor	MCC of Activity Classifiers	MCC of Selectivity Classifiers
5-HT_1B_	0.7867	0.8057
5-HT_2B_	0.7376	0.8238

**Table 5:** All of the interactions considered during the pose filtering phase. The blue cells represent hydrogen bonds, and the red cells represent distance criterion (≤5.0 Å between the compound and any atom of a residue). OBP: orthosteric binding pocket; SBP: secondary binding pocket.

Interactions	1B Crystals	2B Crystals
**OBP**	D129^3.32^	D135^3.32^
	T134^3.37^	S139^3.36^
	S212^5.42^	
**SBP**		Q359^7.32^
	M337^6.58^	M218^5.39^
	V201^ECL2.52^	L209^ECL2.52^
